# Hallux Alignment and Flexor Hallucis Brevis Morphology Are Independently Associated With Jump‐Landing Stability in Adolescent Athletes

**DOI:** 10.1111/sms.70342

**Published:** 2026-07-11

**Authors:** Yasunari Ikuta, Noriaki Maeda, Tsubasa Tashiro, Satoshi Arima, Honoka Ishihara, Sakura Oda, Ayano Ishida, Minoru Toriyama, Hajime Ito, Ryuya Yamakawa, Tomoyuki Nakasa, Yukio Mikami, Nobuo Adachi

**Affiliations:** ^1^ Department of Orthopaedic Surgery Hiroshima University Hospital Hiroshima Japan; ^2^ Sports Medical Center Hiroshima University Hospital Hiroshima Japan; ^3^ Department of Sports Rehabilitation, Graduate School of Biomedical and Health Sciences Hiroshima University Hiroshima Japan; ^4^ Department of Artificial Joints and Biomaterials, Graduate School of Biomedical and Health Sciences Hiroshima University Hiroshima Japan; ^5^ Department of Rehabilitation Medicine Hiroshima University Hospital Hiroshima Japan

**Keywords:** adolescent athletes, dynamic postural stability index, flexor hallucis brevis, hallux valgus, injury prevention, intrinsic foot muscles, skeletal maturity, ultrasonography

## Abstract

The functional impact of hallux valgus (HV) on dynamic postural stability in adolescent athletes remains unclear. We investigated whether hallux alignment and intrinsic foot muscle morphology are associated with dynamic postural stability during a jump‐landing task. This study included 185 adolescent athletes (mean age, 13.6 years). Participants with perceived ankle instability (Cumberland Ankle Instability Tool score ≤ 25 in either ankle) were excluded. Weight‐bearing radiographs were used to measure the HV angle (HVA), hallux interphalangeal angle (HIA), and skeletal maturity. Ultrasonography was used to quantify the cross‐sectional area (CSA) of intrinsic foot muscles, including the flexor hallucis brevis (FHB). The Dynamic Postural Stability Index (DPSI) was assessed during a single‐leg jump‐landing task. Associations were examined using linear mixed‐effects models, accounting for within‐participant correlation across limbs. HV and interphalangeal HV were present in approximately 33% and 86% of feet, respectively. In the multivariable model, greater combined hallux deformity (HVA + HIA) and open physes were independently associated with higher DPSI values, indicating poorer stability. Conversely, larger FHB CSA and higher body mass index (BMI) were associated with lower DPSI values, indicating better stability (all *p* < 0.05). Neither limb side nor the interaction between limb side and hallux alignment was significant. These findings suggest that hallux alignment, FHB morphology, and skeletal maturity are associated with jump‐landing stability in adolescent athletes, without evidence of limb‐dependent effects. Static hallux alignment and intrinsic foot muscle morphology provide clinically relevant information for evaluating dynamic stability in this population.

## 1 Introduction

Hallux valgus (HV) is a common foot deformity among adolescents [1, 2]. In adolescent athletes, HV appears more prevalent in females and in dance disciplines characterized by repetitive forefoot loading, raising concern about sports‐related symptoms and functional limitations [3, 4]. Despite increasing attention to HV in adolescents, its functional implications during dynamic sport‐specific tasks remain unknown. In adults, HV contributes to pain and functional impairment [5], and more severe deformity correlates with increased postural sway [6], poorer balance, and slower gait [7]. HV has also been shown to alter sagittal‐plane hallux kinematics during walking [8]. However, these findings are largely based on older populations and low‐ to moderate‐demand tasks, and their relevance to adolescent athletes performing high‐demand athletic tasks remains to be established.

Balance impairments have also been reported in children with HV and other foot deformities [9]; however, the relationship between HV and postural stability in adolescent athletes has not been fully elucidated. Athletic adolescents represent a distinct population in which postural control continues to mature through age‐related optimization of sensorimotor integration and postural strategies [10]. Sex‐ and age‐related differences are also evident [11], with female adolescents generally demonstrating better postural stability than males [12]. Moreover, sport‐specific training may induce neuromuscular adaptations that modify the functional impact of HV [13]; for example, adolescent ballet dancers with HV show better balance performance than sedentary individuals with HV, potentially attributable to training‐related compensation [14]. Therefore, the functional effect of HV in athletes appears to be context‐dependent rather than uniformly negative. While compensatory neuromuscular adaptations may be adequate for low‐ to moderate‐demand balance tasks, they may be insufficient during high‐demand sport‐specific tasks—such as jump landing—that require rapid impact attenuation and immediate single‐limb restabilization.

Limited research has directly examined how HV relates to dynamic postural stability in adolescent athletes—an age group characterized by high sports participation and active neuromuscular development [10, 12]. This gap is clinically relevant because poorer performance on single‐leg dynamic stability tasks is associated with an increased risk of sports‐related injury in young athletes [15, 16]. From a biomechanical perspective, HV‐related deficits may be particularly relevant during sport‐specific tasks requiring rapid attenuation of impact forces and reestablishment of single‐limb control, such as jump landing [17, 18].

In this context, intrinsic foot muscles contribute to medial longitudinal arch support, attenuation of ground reaction force (GRF), and functional foot stiffness during propulsion [19]. In adults with HV, a reduced cross‐sectional area (CSA) of the abductor hallucis (AbH) and flexor hallucis brevis (FHB) has been reported, suggesting diminished intrinsic muscular support of the first ray–hallux complex [20, 21]. However, whether intrinsic foot muscle morphology is related to dynamic postural stability in adolescent athletes with HV remains unclear.

Therefore, this study primarily aimed to investigate the association between HV deformity and dynamic postural stability during a single‐leg jump‐landing task in adolescent athletes. A secondary aim was to determine whether intrinsic foot muscle morphology is associated with functional stability during dynamic tasks.

## 2 Materials and Methods

### 2.1 Participants

This cross‐sectional study enrolled adolescent athletes who underwent annual medical and physical checkups at the Sports Medical Center of Hiroshima University Hospital from 2021 to 2024. For athletes who attended this program in multiple years, only data from the first visit were included to avoid within‐participant duplication.

A total of 250 athletes (164 males, 86 females; mean age 13.7 ± 1.6 years; range 10–17 years) were initially included. All participants were selected by the Hiroshima City Sports Association for an elite youth development program aimed at enhancing competitive performance. More than 60% of the athletes had competed at national‐level tournaments. Athletes represented a wide range of sports, including archery, badminton, basketball, field hockey, gymnastics, handball, ice hockey, judo, kendo, rugby, sailing, skating, soft tennis, swimming, table tennis, tennis, track and field, and wrestling. The dominant leg was defined as the leg the participant reported using to kick a ball toward a target.

Inclusion criteria were (1) participation in the athlete development program and (2) absence of diagnosed lower extremity musculoskeletal disorders. Exclusion criteria were: (1) any lower limb injury within the preceding 3 months requiring cessation of sports participation; (2) a score ≤ 25 on the Japanese version of the Cumberland Ankle Instability Tool (CAIT) [22] in either ankle to minimize confounding by ankle instability; and (3) technical or procedural errors during data acquisition.

Seven participants met exclusion criterion 1, 55 met criterion 2, and three met criterion 3. Accordingly, data from 185 athletes (121 males, 64 females; mean age 13.6 ± 1.6 years; range 10–17 years) were included in the analysis (Table 1 and Table S1).

**TABLE 1:** Participant characteristics (*n* = 185).

Age (years)	13.6 ± 1.6 (range, 10–17 years)
Sex, *n* (%)	Male 121 (65.4); female 64 (34.6)
Dominant limb, *n* (%)	Left 7 (3.8); right 178 (96.2)
BMI (kg/m^2^)	20.3 ± 3.8 (95% CI, 19.7–20.8)
CAIT score	28.9 ± 1.3 (95% CI, 28.7–29.0)

*Note:* Values are presented as mean ± SD or *n* (%). Parentheses for BMI and CAIT indicate 95% confidence intervals. The dominant limb was defined as the preferred leg used to kick a ball toward a target. BMI, body mass index; CAIT, Cumberland Ankle Instability Tool.

Written informed consent was obtained from all participants and their legal guardians. The study protocol was approved by the Ethical Committee for Epidemiology of Hiroshima University (Approval No. E‐941) and was conducted in accordance with the Declaration of Helsinki.

### 2.2 Radiographic Assessment

Weight‐bearing foot radiographs were obtained in dorsoplantar and lateral views with participants standing in a relaxed posture and distributing weight evenly between both lower limbs. Images were acquired using a digital radiographic system and analyzed with ShadeQuest/ViewR‐DG (version 1.26; Fujifilm Medical Co. Ltd., Tokyo, Japan). From the dorsoplantar view, the following parameters were measured: HV angle (HVA), defined as the angle between the longitudinal axes of the first metatarsal and the proximal phalanx of the hallux; and the hallux interphalangeal angle (HIA), defined as the angle between the longitudinal axes of the proximal and distal phalanges of the hallux. Growth plate (GP) status was determined based on whether the proximal physis of the first metatarsal was closed (GP = 0) or open (GP = 1). From the lateral view, the following parameters were assessed: the lateral talo–first metatarsal angle (Meary angle), defined as the angle between the longitudinal axes of the talus and the first metatarsal, and calcaneal pitch angle (CPA), defined as the angle between the inferior border of the calcaneus and the supporting surface.

For classification purposes, HV was defined as HVA ≥ 15°, and interphalangeal HV was defined as HIA ≥ 10°, in accordance with established criteria [23, 24]. These thresholds were used to determine deformity prevalence in the study. All radiographic measurements were performed by a single experienced examiner who was blinded to clinical and functional outcomes. Measurement definitions and procedures followed previously validated methodologies for radiographic assessment of foot deformities [25, 26, 27, 28].

### 2.3 Ultrasound Imaging

The CSA of three intrinsic foot muscles—the AbH, FHB, and flexor digitorum brevis (FDB)—was measured using B‐mode ultrasonography (Noblus; Hitachi Ltd., Tokyo, Japan) with an 8‐MHz linear‐array transducer. These muscles were selected because of their established contributions to medial longitudinal arch support and dynamic foot function.

Participants were examined in the prone position with the knee flexed to 90° and the ankle maintained in a neutral position. To standardize transducer placement, anatomical landmarks were identified and marked on the skin before image acquisition. Transverse images were obtained at predefined locations as follows: (1) AbH—the probe was placed anterior to the medial malleolus and oriented perpendicular to the longitudinal axis of the foot; (2) FHB—the probe was positioned perpendicular to a line parallel to the muscle belly on the plantar aspect of the midfoot; and (3) FDB—the probe was oriented perpendicular to the line connecting the third toe and the medial calcaneal tuberosity.

Minimal probe pressure was applied to reduce soft‐tissue compression during scanning. For each muscle, CSA was quantified by manually tracing the muscle boundary on a still image using the built‐in analysis software. All measurements were obtained from both the non‐dominant and dominant feet. Each muscle was measured three times, and the mean CSA of the three measurements was used for analysis. Intra‐session variability was quantified using the within‐foot coefficient of variation across the three repeated measurements. The protocol was based on previously validated procedures demonstrating high intra‐rater reliability for ultrasound‐based intrinsic foot muscles morphometry [29].

### 2.4 Muscle Strength Assessment

Isokinetic muscle strength of ankle plantarflexion and dorsiflexion was assessed using a Biodex System 4 dynamometer (Biodex Medical Systems Inc., Shirley, NY, USA) in concentric mode at a constant angular velocity of 60°/s. Participants were seated according to the manufacturer's guidelines, with the hip flexed to approximately 85°. The trunk and thigh were stabilized with harness straps, and the test foot was secured to the footplate using adjustable hook‐and‐loop straps to minimize compensatory movement.

Before data collection, participants completed submaximal practice trials to familiarize themselves with the equipment and testing procedure. They performed three maximal‐effort repetitions of plantarflexion and dorsiflexion with the non‐dominant and dominant limbs. Participants were provided with real‐time visual feedback of torque output on the dynamometer display during testing. A rest interval of at least 60 s was provided between trials to reduce the influence of fatigue. Peak torque (Nm) was recorded for each repetition, and the highest value was expressed as a percentage of body weight (peak torque/body weight, %) for subsequent analysis.

### 2.5 Dynamic Postural Stability Measurement

Dynamic postural stability was assessed using the Dynamic Postural Stability Index (DPSI), derived from GRF data obtained during a standardized single‐leg forward jump‐landing task. The testing procedure was based on previously validated methods [30].

Participants started each trial at a distance corresponding to 40% of body height from the anterior edge of a force platform (49.5 × 49.5 cm; AccuGait, AMTI, Watertown, MA, USA). A 30‐cm hurdle was placed midway between the starting position and the platform. Participants were instructed to jump forward over the hurdle using both feet and land on a single limb, contacting the center of the force plate. After landing, participants were instructed to stabilize as quickly as possible, place both hands on their hips, and maintain a steady posture for at least 10 s while looking straight ahead. Three practice trials were performed to familiarize participants with the task. Test trials were repeated if the participant contacted the hurdle, lost balance, landed outside the platform boundaries, or if the limbs contacted each other during landing. A 60‐s rest interval was provided between trials to minimize fatigue.

GRF signals were sampled at 200 Hz and filtered using a zero‐lag, second‐order Butterworth low‐pass filter with a cutoff frequency of 20 Hz. Initial ground contact was defined as the point at which the vertical GRF exceeded 5% of the participant's body weight (BW). DPSI was computed from GRF variability in the mediolateral (*x*), anteroposterior (*y*), and vertical (*z*) directions during the first 3 s following initial ground contact and normalized to *BW*. In addition to the composite DPSI value, directional indices—mediolateral stability index (MLSI), anteroposterior stability index (APSI), and vertical stability index (VSI)—were calculated using the same time window and normalization approach, as follows:

$$\text{MLSI}=\frac{\sqrt{\sum {\left(0-\mathrm{GR}F_{x}\right)}^{2}/N}}{\mathrm{BW}}$$

$$\text{APSI}=\frac{\sqrt{\sum {\left(0-\mathrm{GR}F_{y}\right)}^{2}/N}}{\mathrm{BW}}$$

$$\mathrm{VSI}=\frac{\sqrt{\sum {\left(\mathrm{BW}-\mathrm{GR}F_{z}\right)}^{2}/N}}{\mathrm{BW}}$$

$$\text{DPSI}=\frac{\sqrt{\left(\sum {\left(0-\mathrm{GR}F_{x}\right)}^{2}+\sum {\left(0-\mathrm{GR}F_{y}\right)}^{2}+\sum {\left(\mathrm{BW}-\mathrm{GR}F_{z}\right)}^{2}\right)/N}}{\mathrm{BW}}$$

where *N* represents the number of data points within the 3‐s post‐landing window

All analyses were performed in MATLAB R2021a (MathWorks Inc., Natick, MA, USA). The mean value from three successful trials was used for statistical analysis.

### 2.6 Statistical Analysis

A single observer (Y.I.) performed all radiographic measurements using a standardized protocol. To assess intra‐rater reliability, the observer repeated measurements on a random subset of 40 ft (10.8%) after an interval of at least 6 weeks, blinded to the initial results. Intra‐rater reliability was calculated using the intraclass correlation coefficient (ICC (3,1)) based on a two‐way mixed‐effects model with absolute agreement for single measurements.

Multiple linear mixed‐effects models (LMM) were used to investigate associations between dynamic postural stability and candidate morphological and biomechanical factors while accounting for within‐participant correlation between limbs. Given functional differences between the non‐dominant and dominant limbs in sport‐related unilateral tasks [31, 32], limb side (non‐dominant vs. dominant) was included as a fixed effect, and a limb‐by‐alignment interaction term was added to examine potential side‐specific associations. To distinguish chronological age from skeletal maturity and reduce multicollinearity between these variables, a GP‐adjusted age residual was obtained by fitting a linear regression model with age as the dependent variable and GP status as the independent variable, and saving the residuals. Both GP status and GP‐adjusted age residual were included as fixed effects in the mixed‐effects model.

Because CPA and Meary angle capture overlapping aspects of sagittal foot alignment, CPA was primarily used for better measurement reproducibility [28]. As a sensitivity analysis, the mixed‐effects model was refitted after replacing CPA with the Meary angle. The DPSI served as the dependent variable. Fixed effects included body mass index (BMI), sex, radiographic parameters (sum of HVA and HIA [HVA + HIA] and CPA), isokinetic ankle strength (plantarflexion and dorsiflexion, peak torque/body weight, %), CSA of intrinsic foot muscles (AbH, FHB, and FDB), GP status (0 = closed physes, 1 = open physes), and GP‐adjusted age residual. All fixed‐effect predictors were entered simultaneously. Repeated measurements from the non‐dominant and dominant limbs were modeled with participants as a random effect to account for within‐participant correlation. The limb side was treated as a repeated factor, assuming a compound symmetry covariance structure.

Additional sensitivity and exploratory analyses were performed. To assess the potential impact of excluding participants with low CAIT scores, participants included in the primary analysis were compared with those excluded due to low CAIT scores. Pearson's correlation analysis was used to examine the association between participant‐level mean HVA + HIA and FHB CSA, calculated by averaging values across the non‐dominant and dominant limbs. The primary LMM was repeated using APSI, MLSI, and VSI as exploratory dependent variables. To further evaluate the effect of CAIT‐based exclusion, a sensitivity LMM was retained for participants with low CAIT scores and added CAIT score as a covariate. To address heterogeneity in sport disciplines and familiarity with landing tasks, another sensitivity LMM was restricted to athletes participating in sports involving frequent jumping, landing, cutting, or rapid deceleration. These sports included badminton, basketball, field hockey, gymnastics, handball, rugby, soft tennis, tennis, track and field, and wrestling.

Multicollinearity was assessed using the variance inflation factor (VIF) computed from the fixed‐effect design matrix, with values > 10 indicating potential multicollinearity. Statistical significance was set at *p* < 0.05. All analyses were performed using IBM SPSS Statistics for Windows, version 27.0 (IBM, Armonk, NY, USA).

## 3 Results

Intra‐rater reliability testing in 40 ft demonstrated good‐to‐excellent reliability across all radiographic parameters (ICCs, 0.888–0.971), supporting single‐rater measurements for the primary analyses (Table S2).

Participants excluded because of low CAIT scores had substantially lower CAIT scores than those included in the primary analysis. Other key variables, including age, sex, BMI, GP status, HVA, HIA, HVA + HIA, FHB CSA, and DPSI, did not differ significantly between groups (Table S3).

Weight‐bearing radiographs demonstrated a high prevalence of hallux alignment abnormalities. HV (HVA ≥ 15°) was identified in 57 non‐dominant feet (30.8%) and 66 dominant feet (35.7%). Similarly, interphalangeal HV (HIA ≥ 10°) was observed in 159 non‐dominant feet (86.0%) and 158 dominant feet (85.4%). All radiographic alignment variables are shown in Table 2. Ultrasound imaging provided CSA measurements of intrinsic foot muscles (AbH, FHB, and FDB). The mean intra‐session coefficients of variation for the three repeated CSA measurements were 1.23% ± 2.14% for AbH, 1.35% ± 2.30% for FHB, and 1.16% ± 1.13% for FDB. Additionally, isokinetic testing quantified ankle plantarflexion and dorsiflexion strength as peak torque/body weight (%). These measures are summarized in Table 2.

**TABLE 2:** Radiographic parameters, muscle strength, intrinsic foot muscle morphology, and dynamic postural stability in non‐dominant and dominant limbs.

Variable	Non‐dominant limb (*n* = 185)	Dominant limb (*n* = 185)
HVA (°)	12.9 ± 4.6 (12.3–13.6)	13.4 ± 4.7 (12.7–14.1)
HIA (°)	14.0 ± 3.8 (13.4–14.5)	13.5 ± 3.7 (12.9–14.0)
HVA + HIA (°)	26.9 ± 6.1 (26.0–27.8)	26.9 ± 5.8 (26.0–27.7)
Meary angle (°)	2.3 ± 6.8 (1.4–3.3)	3.0 ± 6.8 (2.0–4.0)
CPA (°)	18.6 ± 4.6 (17.9–19.2)	18.1 ± 4.7 (17.5–18.8)
Growth plate status (open/closed), *n* (%)	Open 74 (40), closed 111 (60)	Open 74 (40), closed 111 (60)
Ankle plantarflexion strength, peak torque/body weight (%)	85.4 ± 31.7 (80.8–90.0)	92.4 ± 33.2 (87.6–97.2)
Ankle dorsiflexion strength, peak torque/body weight (%)	37.8 ± 10.5 (36.2–39.3)	40.1 ± 13.5 (38.2–42.1)
AbH CSA (mm^2^)	209.5 ± 60.5 (200.7–218.2)	216.5 ± 57.2 (208.2–224.8)
FHB CSA (mm^2^)	204.6 ± 55.2 (196.6–212.6)	210.9 ± 61.4 (202.0–219.8)
FDB CSA (mm^2^)	190.0 ± 57.7 (181.6–198.3)	192.6 ± 56.2 (184.4–200.7)
DPSI	0.306 ± 0.038 (0.300–0.311)	0.306 ± 0.037 (0.301–0.312)
APSI	0.136 ± 0.011 (0.134–0.137)	0.135 ± 0.010 (0.133–0.136)
MLSI	0.030 ± 0.007 (0.029–0.031)	0.030 ± 0.007 (0.029–0.031)
VSI	0.272 ± 0.041 (0.266–0.278)	0.273 ± 0.039 (0.267–0.278)

*Note:* Values are presented as mean ± SD (95% confidence interval) or *n* (%). The dominant limb was defined as the preferred leg used to kick a ball toward a target.

Abbreviations: AbH, abductor hallucis; APSI, anteroposterior stability index; CPA, calcaneal pitch angle; CSA, cross‐sectional area; DPSI, dynamic postural stability index; FDB, flexor digitorum brevis; FHB, flexor hallucis brevis; HIA, hallux interphalangeal angle; HVA, hallux valgus angle; MLSI, mediolateral stability index; VSI, vertical stability index.

**FIGURE 1:**
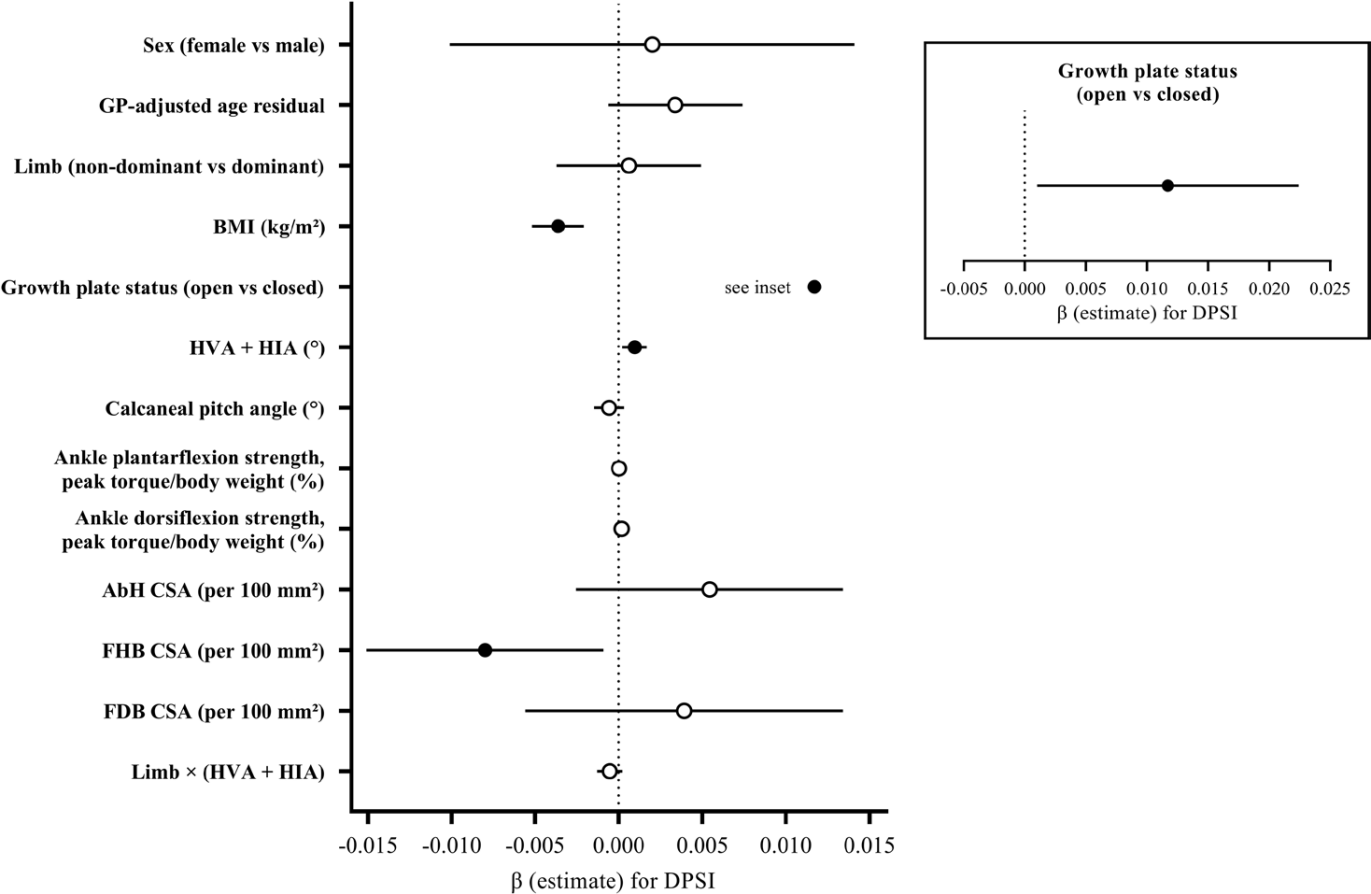
Forest plot of fixed‐effect estimates (β) with 95% confidence intervals for the Dynamic Postural Stability Index (DPSI). Filled circles indicate statistical significance (*p* < 0.05); open circles indicate non‐significance (*p* ≥ 0.05). Horizontal lines represent 95% confidence intervals. For binary predictors, β represents the adjusted difference in the Dynamic Postural Stability Index relative to the reference group. The inset panel displays the estimate for growth plate status on an expanded scale, as its wide confidence interval exceeds the range of the main panel. Reference categories were: Male (Sex), Dominant (Limb side), and Closed (Growth plate status). GP, growth plate; BMI, body mass index; HVA, hallux valgus angle; HIA, hallux interphalangeal angle; AbH, abductor hallucis; CSA, cross‐sectional area; FHB, flexor hallucis brevis; FDB, flexor digitorum brevis.

An LMM was used to examine associations between dynamic postural stability and candidate factors while accounting for within‐participant correlation between limbs. HVA + HIA, GP status, BMI, and FHB CSA were independently associated with DPSI (*p* < 0.05) (Figure 1, Table 3). Greater combined hallux deformity (higher HVA + HIA) and open physes were associated with higher DPSI values (poorer stability), whereas higher BMI and larger FHB CSA were associated with lower DPSI values (better stability). The GP‐adjusted age residual was not significant (*p* = 0.098). Limb side, sex, CPA, ankle plantarflexion and dorsiflexion strength, and CSA of AbH and FDB were not significant. The limb‐by‐HVA + HIA interaction was not significant (*p* = 0.161), indicating no evidence that the association between hallux alignment and DPSI differed by limb side. Multicollinearity was not problematic (all VIFs < 2.2). At the participant level, mean HVA + HIA was weakly positively correlated with mean FHB CSA (*r* = 0.18, *p* = 0.013). Exploratory analyses using directional stability indices as outcomes revealed partially direction‐specific associations. HVA + HIA, BMI, GP status, and FHB CSA were independently associated with VSI; BMI was associated with APSI; and FHB CSA and CPA were associated with MLSI. The limb‐by‐HVA + HIA interaction was not significant for any directional index (Table S4).

**TABLE 3:** Fixed effects from the linear mixed‐effects model predicting DPSI (*n* = 185 participants; 370 limb observations).

Predictor	*β* (estimate)	95% CI	*p* value
Sex (female vs. male)	0.00201	−0.0101, 0.0141	0.744
GP‐adjusted age residual	0.00338	−0.000628, 0.00740	0.098
Limb (non‐dominant vs. dominant)	0.000609	−0.00371, 0.00493	0.781
BMI (kg/m^2^)	−0.00363	−0.00518, −0.00208	< 0.001*
Growth plate status (open vs. closed)	0.0117	0.000996, 0.0224	0.032*
HVA + HIA (°)	0.000947	0.000211, 0.00168	0.030*
CPA (°)	−0.000575	−0.00147, 0.000321	0.208
Ankle plantarflexion strength, peak torque/body weight (%)	0.0000102	−0.000127, 0.000147	0.884
Ankle dorsiflexion strength, peak torque/body weight (%)	0.000181	−0.000174, 0.000536	0.317
AbH CSA (per 100 mm^2^)	0.00544	−0.00256, 0.0134	0.182
FHB CSA (per 100 mm^2^)	−0.00799	−0.0151, −0.000920	0.027*
FDB CSA (per 100 mm^2^)	0.00391	−0.00558, 0.0134	0.419
Limb × (HVA + HIA)	−0.000536	−0.00129, 0.000215	0.161

*Note:* Linear mixed‐effects model with participants as a random effect and limb side as a repeated measure. GP‐adjusted age residual was defined as the residuals from a linear regression model with age as the dependent variable and GP status as the independent variable. Coding: Growth plate (0 = closed, 1 = open); HVA + HIA (sum of hallux valgus and interphalangeal angles, mean‐centered). CSA coefficients correspond to a 100 mm^2^ increase. *p* values are from Type III tests of fixed effects.

Abbreviations: AbH, abductor hallucis; BMI, body mass index; CPA, calcaneal pitch angle; CSA, cross‐sectional area; FDB, flexor digitorum brevis; FHB, flexor hallucis brevis; GP, growth plate; HIA, hallux interphalangeal angle; HVA, hallux valgus angle.

* *p* < 0.05.

Given its better measurement reproducibility, CPA was selected for the primary model, whereas the Meary angle was examined in a sensitivity analysis that yielded results similar to the primary model. HVA + HIA, BMI, FHB CSA, and GP status remained independently associated with DPSI (all *p* < 0.05), while other variables remained nonsignificant. The limb‐by‐HVA + HIA interaction also remained nonsignificant, indicating that the association between hallux alignment and DPSI did not differ by limb side in this sensitivity model.

In a sensitivity analysis retaining participants with low CAIT scores and additionally adjusting for CAIT score, HVA + HIA, BMI, and GP status remained independently associated with DPSI, whereas CAIT score itself was not significant. The GP‐adjusted age residual was also associated with DPSI in this model. No significant limb‐by‐HVA + HIA interaction was observed. Although the association between FHB CSA and DPSI was attenuated and was not statistically significant, the direction of the estimate remained consistent with the primary model (Table S5).

In a sensitivity analysis restricted to athletes in sports involving frequent jumping, landing, cutting, pivoting, or rapid deceleration (*n* = 97; 194 limb observations), BMI, FHB CSA, and GP status remained independently associated with DPSI. Additionally, the GP‐adjusted age residual and ankle dorsiflexion strength were associated with DPSI in this subset. Although the association between HVA + HIA and DPSI was attenuated and was not statistically significant, the direction of the estimate remained consistent with the primary model. The limb‐by‐HVA + HIA interaction remained nonsignificant (Table S6).

## 4 Discussion

In this cross‐sectional study of adolescent athletes, we examined whether hallux valgus–related alignment and intrinsic foot muscle morphology were associated with dynamic postural stability during a single‐leg jump‐landing task. Using an LMM that included both limbs and accounted for within‐participant correlation between limbs, we found that HVA + HIA, GP status, BMI, and FHB CSA were independently associated with DPSI. Specifically, greater combined HV deformity and open physes were associated with higher DPSI values (indicating poorer stability), whereas higher BMI and larger FHB CSA were associated with lower DPSI values (indicating better stability). Neither limb side nor the limb‐by‐HVA + HIA interaction was significant, indicating no evidence of limb‐dependent effects. Additional analyses refined these findings: the HVA + HIA association was robust when participants with low CAIT scores were retained but was attenuated in the smaller landing‐relevant sports subset, whereas FHB CSA showed consistent associations in the primary model, directional analyses, and landing‐relevant sports subset, although it was attenuated when participants with low CAIT scores were retained. GP status was the most consistent maturity‐related factor, while the GP‐adjusted age residual emerged only in sensitivity analyses. Overall, these findings suggest that hallux alignment, FHB morphology, and skeletal maturity are associated with dynamic postural stability in adolescent athletes. However, these associations may vary according to perceived ankle instability status and sport‐specific context.

Dynamic postural stability during landing is clinically relevant, as poorer single‐limb dynamic balance performance is a known risk factor for lower extremity injury in young athletes [15, 16]. In this context, the independent associations of HVA + HIA, FHB morphology, BMI, and GP status with DPSI highlight that both alignment and neuromuscular capacity contribute to landing stabilization demands. Our finding that greater combined angular deformity (HVA + HIA) was associated with higher DPSI values is consistent with reports of HV‐related balance impairment and altered postural control across various age groups [5, 6, 7, 9]. Previous studies have shown inconsistent associations between HV and postural stability, which may be attributable to differences in testing protocols, symptom severity, and population characteristics [5, 33]. Methodologically, excluding participants with a low CAIT score in either limb effectively minimized confounding from ankle instability [34], facilitating the isolation of the specific association between hallux alignment and DPSI. Specifically, HV‐related deficits may be difficult to detect during low‐demand tasks such as static standing or level walking, but may become pronounced during high‐demand tasks such as jump landing that require rapid impact absorption and single‐limb restabilization [5, 18]. Notably, despite reported functional specialization between non‐dominant and dominant limbs in sport‐related unilateral tasks [31, 32], we found no evidence of limb‐dependent effects: neither limb side nor the limb‐by‐HVA + HIA interaction was significant. This is consistent with findings that dynamic stability scores often remain similar between limbs despite potential strategic differences [18]. This suggests that, within this landing task, hallux alignment affects stability mechanics across both limbs. In athletic adolescents, sport‐specific neuromuscular adaptations may partially compensate for HV‐related biomechanical disadvantages, potentially attenuating observable deficits under certain conditions [14]. Therefore, the functional impact of HV in this population should be interpreted in relation to task demands, symptoms, and the athlete's developmental background. The model coefficient also provides context for practical relevance when translated into an interpretable scale. Based on this coefficient, a 10° greater HVA + HIA corresponds to an approximately 0.0095 higher DPSI, which is about one‐quarter of the observed SD of DPSI in this cohort. Although this magnitude is modest, it may be meaningful because landing stability is shaped by multiple interacting biomechanical and neuromuscular factors. Nevertheless, given the absence of an established DPSI threshold for injury risk in adolescent athletes, this estimate should be interpreted in relation to the DPSI distribution in the present cohort rather than as a definitive clinical cutoff.

Exploratory directional analyses provided further mechanistic insights. HVA + HIA was associated with VSI but not with APSI or MLSI, suggesting that the association between hallux alignment and composite DPSI was mainly observed in the vertical stability component. In contrast, FHB CSA was associated with both VSI and MLSI. The association with VSI is consistent with a previous study suggesting that FHB CSA may contribute to vertical force attenuation during single‐leg landing in adults [35]. The association with MLSI extends this concept and raises the possibility that FHB morphology may also affect mediolateral stabilization by supporting the first metatarsophalangeal joint and enhancing medial column stiffness during rapid load acceptance. These partially distinct directional patterns suggest that hallux alignment and intrinsic muscle morphology may contribute to dynamic stability through different, although overlapping, biomechanical pathways.

A key novel finding of this study is that a larger FHB CSA is independently associated with better dynamic postural stability during landing. Intrinsic foot muscles contribute to medial longitudinal arch support, regulation of foot stiffness, and attenuation of GRF during stance and propulsion [19, 36, 37]. Biomechanically, the FHB stabilizes the first metatarsophalangeal joint and modulates hallux loading during the rapid stabilization phase of landing. Consequently, a larger FHB CSA may indicate greater intrinsic capacity to support the medial column under high‐demand conditions, potentially counteracting the destabilizing effects of hallux malalignment. This interpretation is consistent with reports of reduced FHB size in adults with HV and evidence relating intrinsic muscle morphology to foot mechanics and performance‐related outcomes [21, 35]. The participant‐level mean HVA + HIA was only weakly and positively correlated with the mean FHB CSA. This finding, along with the low VIF values in the mixed‐effects model, supports the interpretation that FHB CSA was not a surrogate for hallux deformity severity but provided independent information in the model. Taken together, these findings suggest that intrinsic hallux flexor morphology represents a biologically plausible and potentially modifiable contributor to dynamic postural stability in adolescent athletes.

The association between open physes and higher DPSI may represent more than incomplete neuromuscular maturation. Open physes also indicate that the foot is still undergoing structural development, and immature bony and soft‐tissue properties may contribute to less predictable responses to landing loads. The nonsignificant GP‐adjusted age residual in the primary model suggests that physeal status provided maturity‐related information not captured by chronological age alone. However, GP status should be interpreted as a practical rather than comprehensive marker of biological maturation.

BMI also demonstrated an independent association with DPSI, with higher BMI values corresponding to lower DPSI values (indicating better dynamic stability). However, this finding warrants cautious interpretation. Given that DPSI is normalized to body weight, BMI may capture broadly interrelated factors such as lean muscle mass, strength, and movement proficiency rather than body mass alone. The independent association of skeletal maturity with DPSI in our model suggests that physical growth and neuromuscular development occur concurrently during this period. From a biomechanical perspective, effective jump‐landing stabilization depends on coordinated impact attenuation and rapid control of limb and trunk motion [17]; athletes with higher BMI, potentially reflecting greater physical development, may adopt landing strategies that reduce normalized GRF variability, even if absolute impact forces are higher [13, 18]. Therefore, the observed relationship should not be interpreted as a protective effect of body mass itself. Instead, it suggests that BMI reflects physical maturation and task‐specific mechanics that facilitate normalized stabilization. Fundamentally, landing stability in adolescent athletes is multifactorial, and BMI should be viewed as a contributing correlate rather than a sole determinant. Other factors, such as broader neuromuscular control and hindfoot alignment, also contribute to postural control and may modify landing mechanics [34, 38, 39]. Consistent with this multifactorial perspective, the sensitivity analysis restricted to landing‐relevant sports indicated that athletic background may shape the relationships between foot‐related factors and landing stability. In this subset, the association between HVA + HIA and DPSI was attenuated, whereas the magnitude of the FHB CSA association increased. This contrast may suggest that, among athletes regularly exposed to jumping, landing, cutting, or rapid deceleration, intrinsic foot muscle morphology is more closely related to landing stabilization than static hallux alignment, possibly because sport‐specific adaptations and task familiarity may partially compensate for alignment‐related disadvantages. Ankle dorsiflexion strength was also positively associated with DPSI in this subset. Because this direction indicates poorer stability with greater dorsiflexion strength, and because concentric isokinetic strength does not directly assess eccentric dorsiflexor control during landing, this exploratory finding should be interpreted cautiously. Rather than indicating a protective effect, it may be attributable to sport‐specific landing demands, training exposure, or neuromuscular adaptations. These findings suggest that the relative contributions of hallux alignment, intrinsic foot muscle morphology, and ankle strength to landing stability may vary according to sport‐specific loading demands.

Clinically, these findings highlight potentially modifiable factors that may enhance landing stability in adolescent athletes with hallux malalignment. In particular, the independent association between larger FHB CSA and better dynamic postural stability suggests that intrinsic hallux flexor capacity plays an important role in functional stabilization during landing. Accordingly, interventions targeting intrinsic foot muscle function—specifically exercises emphasizing hallux flexor activation—warrant investigation for prospective evaluation in this population, given their proposed contribution to medial arch support and functional foot stiffness [36]. Furthermore, since hallux alignment (HVA + HIA) was also independently associated with DPSI, clinicians should consider monitoring hallux alignment and symptoms when evaluating dynamic stability and determining training needs. Ultimately, an integrated approach incorporating foot‐specific conditioning (e.g., intrinsic hallux flexor strengthening) and task‐specific landing technique training warrants further investigation [40, 41].

This study has some limitations. First, the cross‐sectional design prevents causal inference regarding the directional relationship between hallux malalignment, muscle morphology, and landing stability. Second, the recruitment of elite athletes from a single center and the exclusion of participants with perceived ankle instability may limit generalizability to recreational populations or those with concomitant ankle pathology. The association between FHB CSA and DPSI was attenuated and was not statistically significant when participants with low CAIT scores were retained, although the direction of the estimate remained consistent with the primary model. Therefore, the FHB‐related finding may be less generalizable to athletes with perceived ankle instability and should be interpreted with caution. Third, the participants represented 18 sport disciplines with different loading requirements and varying familiarity with single‐leg landing tasks. Although a sensitivity analysis restricted to landing‐relevant sports was performed, the attenuation of the HVA + HIA association in this smaller subset suggests that sport‐specific demands may have influenced the observed associations. Larger sport‐specific studies with detailed exposure metrics are warranted. Fourth, DPSI provides a global measure of stability without isolating kinematic mechanisms. As DPSI is normalized to body weight, the inverse association between BMI and DPSI may partly represent a mathematical consequence of the normalization procedure rather than a true protective effect of higher body mass or physical maturation. Accordingly, the BMI‐related finding should be interpreted with caution. Although the jump distance was scaled to body height and participants completed practice trials before data collection, trial repetition rates were not systematically recorded. Therefore, we could not determine whether task success or task difficulty differed across age groups. Fifth, ultrasound‐based CSA assessment has inherent limitations, as intrinsic foot muscles are small, anatomically variable, and require manual tracing of muscle borders. Although a standardized protocol was used—averaging three repeated measurements with low intra‐session variability—the relatively large SDs of some CSA measures suggest substantial inter‐individual variability and potential residual measurement variability. Consequently, CSA should be interpreted as a morphological surrogate rather than a direct measure of intrinsic muscle strength or activation. Finally, all radiographic measurements were performed by a single observer, and inter‐rater reliability was not assessed. Although intra‐rater reliability was good to excellent, the absence of inter‐rater reliability data may limit the generalizability of the radiographic measurements, particularly because HVA + HIA was a central explanatory variable in the present study.

In conclusion, HV–related deformity is independently associated with impaired dynamic postural stability in adolescent athletes, regardless of limb dominance. Larger FHB CSA and skeletal maturity‐related factors were also associated with DPSI, supporting a multifactorial view of landing stability involving static alignment, intrinsic foot muscle morphology, and developmental factors. Future prospective and interventional studies are warranted to determine whether targeted strategies, particularly those emphasizing intrinsic hallux flexor function, can improve landing biomechanics or dynamic stability in adolescent athletes with hallux malalignment.

## 5 Perspective

Future studies should clarify how adolescent hallux valgus, intrinsic foot muscle morphology, and skeletal maturation interact with sport‐specific loading to influence landing mechanics, symptoms, and injury risk over time. Longitudinal designs incorporating three‐dimensional motion analysis, electromyography, repeated ultrasound assessments, and detailed sport‐exposure metrics would help elucidate the mechanisms underlying the relationship between foot structure and dynamic control. Interventional studies are also warranted to determine whether targeted intrinsic foot muscle training, particularly exercises emphasizing hallux flexor function, can improve landing biomechanics or reduce instability‐related risk. Studies in sport‐specific cohorts and in adolescent athletes with ankle instability may further improve the clinical relevance and generalizability of future screening and prevention strategies.

## Funding

The authors have nothing to report.

## Ethics Statement

The study protocol was approved by the Ethical Committee for Epidemiology of Hiroshima University (Approval No. E‐941) and was conducted in accordance with the Declaration of Helsinki.

## Consent

Written informed consent was obtained from all participants and their legal guardians.

## Conflicts of Interest

The authors declare no conflicts of interest.

## Supporting information


**Table S1:** Sports disciplines of participants.

**Table S2:** Intra‐rater reliability of radiographic parameters (*n* = 40 ft).

**Table S3:** Characteristics of participants included in the final analysis and those excluded due to low CAIT scores.

**Table S4:** Exploratory linear mixed‐effects models for directional stability indices.

**Table S5:** Sensitivity analysis retaining participants with low CAIT scores.

**Table S6:** Sensitivity analysis restricted to athletes in jump‐landing relevant sports (*n* = 97 participants; 194 limb observations).

## Data Availability

The data that support the findings of this study are available from the corresponding author upon reasonable request.
