# Supplementary material for: Hallux Alignment and Flexor Hallucis Brevis Morphology Are Independently Associated With Jump‐Landing Stability in Adolescent Athletes
Source: Scand J Med Sci Sports. 2026 Jul 11;36(7):e70342. doi: 10.1111/sms.70342 (PMC13354965; doi:10.1111/sms.70342)
Supplement: Supplementary file 1 — Table S1: Sports disciplines of participants. [file SMS-36-e70342-s005.docx]

**Supplementary Table S1.** Sports disciplines of participants

| **Sport discipline** | **n (%)** |
| --- | --- |
| Archery | 12 (6.5) |
| Badminton | 3 (1.6) |
| Basketball | 9 (4.9) |
| Field hockey | 15 (8.1) |
| Gymnastics | 6 (3.2) |
| Handball | 7 (3.8) |
| Ice hockey | 5 (2.7) |
| Judo | 6 (3.2) |
| Kendo | 16 (8.7) |
| Rugby | 23 (12.4) |
| Sailing | 11 (6.0) |
| Skating | 11 (6.0) |
| Soft tennis | 11 (6.0) |
| Swimming | 16 (8.7) |
| Table tennis | 11 (6.0) |
| Tennis | 10 (5.5) |
| Track and field | 6 (3.2) |
| Wrestling | 7 (3.8) |
| **Total** | **185 (100)** |

Values are number of participants (%).
