# Supplementary material for: Hallux Alignment and Flexor Hallucis Brevis Morphology Are Independently Associated With Jump‐Landing Stability in Adolescent Athletes
Source: Scand J Med Sci Sports. 2026 Jul 11;36(7):e70342. doi: 10.1111/sms.70342 (PMC13354965; doi:10.1111/sms.70342)
Supplement: Supplementary file 2 — Table S2: Intra‐rater reliability of radiographic parameters (n = 40 ft). [file SMS-36-e70342-s003.docx]

**Supplementary Table S2.** Intra-rater reliability of radiographic parameters (n = 40 feet)

| Radiographic parameters | Intraclass correlation | 95% Confidence intervals |
| --- | --- | --- |
| HVA (°) | 0.971 | 0.946–0.984 |
| HIA (°) | 0.888 | 0.798–0.939 |
| Meary angle (°) | 0.912 | 0.839–0.952 |
| CPA (°) | 0.939 | 0.889–0.967 |

ICC values are reported as ICC (3,1) with 95% confidence intervals (two-way mixed-effects model, absolute agreement, single measurements).
