# Supplementary material for: Hallux Alignment and Flexor Hallucis Brevis Morphology Are Independently Associated With Jump‐Landing Stability in Adolescent Athletes
Source: Scand J Med Sci Sports. 2026 Jul 11;36(7):e70342. doi: 10.1111/sms.70342 (PMC13354965; doi:10.1111/sms.70342)
Supplement: Supplementary file 3 — Table S3: Characteristics of participants included in the final analysis and those excluded due to low CAIT scores. [file SMS-36-e70342-s001.docx]

**Supplementary Table S3. Characteristics of participants included in the final analysis and those excluded due to low CAIT scores**

| **Variable** | **Included (n = 185)** | **Excluded due to low CAIT (n = 55)** | **P value** |
| --- | --- | --- | --- |
| Age (years) | 13.6 ± 1.6 (range, 10–17 years) | 13.6 ± 1.6 (range, 10–17 years) | 0.83 |
| Sex, n (%) | male 121 (65.4); female 64 (34.6) | male 35 (63.6); female 20 (36.4) | 0.81 |
| BMI (kg/m^2^) | 20.3 ± 3.8 (95% CI, 19.7–20.8) | 20.6 ± 4.4 (95% CI, 19.4–21.8) | 0.58 |
| Minimum CAIT score | 28.9 ± 1.3 (95% CI, 28.7–29.0) | 18.4 ± 7.9 (95% CI, 16.2–20.5) | <0.001 |
| HVA (°) | 13.1 ± 4.6 (12.7–13.6) | 13.9 ± 5.5 (12.9–15.0) | 0.17 |
| HIA (°) | 13.7 ± 3.8 (13.3–14.1) | 14.2 ± 3.5 (13.5–14.8) | 0.26 |
| HVA + HIA (°) | 26.9 ± 5.9 (26.3–27.5) | 28.1 ± 6.3 (26.9–29.3) | 0.072 |
| Growth plate status (open/ closed), n (%) | open 74 (40), closed 111 (60) | open 22 (40), closed 33 (60) | 1.00 |
| FHB CSA (mm²) | 207.8 ± 58.4 (201.8–213.7) | 200.7 ± 60.2 (189.4–212.1) | 0.28 |
| DPSI | 0.306 ± 0.038 (0.302–0.310) | 0.309 ± 0.041 (0.302–0.317) | 0.47 |

Values are presented as mean ± SD (95% confidence interval) or n (%). BMI, body mass index; CAIT, Cumberland Ankle Instability Tool; HVA, hallux valgus angle; HIA, hallux interphalangeal angle; FHB, flexor hallucis brevis; CSA, cross-sectional area; DPSI, dynamic postural stability index.

For limb-specific variables, values were averaged across the dominant and non-dominant limbs for each participant before group comparison. Minimum CAIT score was used because exclusion was based on a low score in either ankle.

Between-group comparisons were performed using Welch’s t-test for continuous variables and chi-square test for categorical variables.
