# Supplementary material for: Hallux Alignment and Flexor Hallucis Brevis Morphology Are Independently Associated With Jump‐Landing Stability in Adolescent Athletes
Source: Scand J Med Sci Sports. 2026 Jul 11;36(7):e70342. doi: 10.1111/sms.70342 (PMC13354965; doi:10.1111/sms.70342)
Supplement: Supplementary file 4 — Table S4: Exploratory linear mixed‐effects models for directional stability indices. [file SMS-36-e70342-s004.docx]

**Supplementary Table S4. Exploratory linear mixed-effects models for directional stability indices.**

| **Predictor** | **APSI β (95% CI)** | **p value** | **MLSI β (95% CI)** | **p value** | **VSI β (95% CI)** | **p value** |
| --- | --- | --- | --- | --- | --- | --- |
| Sex (female vs male) | 0.000303 (−0.00322, 0.00383) | 0.865 | −0.000742 (−0.00335, 0.00186) | 0.575 | 0.00228 (−0.0106, 0.0152) | 0.728 |
| GP-adjusted age residual | 0.00105 (−0.000116, 0.00221) | 0.077 | 0.0000915 (−0.000777, 0.000960) | 0.836 | 0.00330 (−0.000979, 0.00757) | 0.130 |
| Limb (non-dominant vs dominant) | 0.000992 (−0.000375, 0.00236) | 0.154 | 0.00000544 (−0.000643, 0.000654) | 0.987 | 0.000165 (−0.00447, 0.00479) | 0.944 |
| BMI (kg/m²) | −0.000511 (−0.000963, −0.0000597) | 0.027* | −0.00000129 (−0.000329, 0.000327) | 0.994 | −0.00385 (−0.00550, −0.00220) | <0.001* |
| Growth plate status (open vs closed) | 0.00197 (−0.00113, 0.00507) | 0.212 | −0.000156 (−0.00248, 0.00217) | 0.895 | 0.0123 (0.000870, 0.0236) | 0.035* |
| HVA + HIA (°) | 0.000148 (−0.0000784, 0.000374) | 0.182 | 0.0000278 (−0.0000940, 0.000150) | 0.711 | 0.000998 (0.000210, 0.00179) | 0.033* |
| CPA (°) | 0.000100 (−0.000167, 0.000367) | 0.461 | −0.000212 (−0.000379, −0.0000453) | 0.013* | −0.000676 (−0.00163, 0.000280) | 0.165 |
| Ankle plantarflexion strength, peak torque/body weight (%) | −0.00000142 (−0.0000410, 0.0000408) | 0.995 | 0.0000110 (−0.0000140, 0.0000360) | 0.387 | 0.0000104 (−0.000136, 0.000157) | 0.888 |
| Ankle dorsiflexion strength, peak torque/body weight (%) | 0.00000609 (−0.000101, 0.000113) | 0.911 | −0.00000830 (−0.0000709, 0.0000543) | 0.794 | 0.000191 (−0.000188, 0.000571) | 0.321 |
| AbH CSA (per 100 mm²) | 0.000982 (−0.00144, 0.00340) | 0.425 | 0.000322 (−0.00108, 0.00172) | 0.650 | 0.00576 (−0.00279, 0.0143) | 0.186 |
| FHB CSA (per 100 mm²) | −0.00130 (−0.00340, 0.000819) | 0.229 | −0.00183 (−0.00313, −0.000527) | 0.006* | −0.00800 (−0.0156, −0.000454) | 0.038* |
| FDB CSA (per 100 mm²) | 0.000827 (−0.00203, 0.00368) | 0.569 | −0.00115 (−0.00284, 0.000537) | 0.181 | 0.00409 (−0.00604, 0.0142) | 0.428 |
| Limb × (HVA + HIA) | −0.0000433 (−0.000281, 0.000195) | 0.720 | −0.0000159 (−0.000127, 0.0000954) | 0.778 | −0.000574 (−0.00138, 0.000232) | 0.162 |

Values are fixed-effect estimates (β) with 95% confidence intervals from exploratory linear mixed-effects models using APSI, MLSI, or VSI as the dependent variable. The same fixed effects and repeated structure as the primary DPSI model were used.

Coding: Growth plate (0 = closed, 1 = open); HVA + HIA (sum of hallux valgus and interphalangeal angles, mean-centered). CSA coefficients correspond to a 100 mm² increase. P values are from Type III tests of fixed effects. *p < 0.05.

APSI, anteroposterior stability index; MLSI, mediolateral stability index; VSI, vertical stability index; GP, growth plate; BMI, body mass index; HVA, hallux valgus angle; HIA, hallux interphalangeal angle; CPA, calcaneal pitch angle; AbH, abductor hallucis; CSA, cross-sectional area; FHB, flexor hallucis brevis; FDB, flexor digitorum brevis.
