# Supplementary material for: Hallux Alignment and Flexor Hallucis Brevis Morphology Are Independently Associated With Jump‐Landing Stability in Adolescent Athletes
Source: Scand J Med Sci Sports. 2026 Jul 11;36(7):e70342. doi: 10.1111/sms.70342 (PMC13354965; doi:10.1111/sms.70342)
Supplement: Supplementary file 6 — Table S6: Sensitivity analysis restricted to athletes in jump‐landing relevant sports (n = 97 participants; 194 limb observations). [file SMS-36-e70342-s006.docx]

**Supplementary Table S6. Sensitivity analysis restricted to athletes in jump-landing relevant sports (n = 97 participants; 194 limb observations)**

| **Predictor** | **β (estimate)** | **95% CI** | **p value** |
| --- | --- | --- | --- |
| Sex (female vs male) | −0.00341 | −0.0209, 0.0141 | 0.701 |
| GP-adjusted age residual | 0.00652 | 0.000347, 0.0127 | 0.039* |
| Limb (non-dominant vs dominant) | −0.00312 | −0.00932, 0.00308 | 0.320 |
| BMI (kg/m²) | −0.00494 | −0.00758, −0.00230 | <0.001* |
| Growth plate status (open vs closed) | 0.0188 | 0.00243, 0.0352 | 0.025* |
| HVA+HIA (°) | 0.000881 | −0.000271, 0.00203 | 0.177 |
| Calcaneal pitch angle (°) | −0.000111 | −0.00132, 0.00110 | 0.856 |
| Ankle plantarflexion strength, peak torque/body weight (%) | −0.000143 | −0.000357, 0.0000710 | 0.175 |
| Ankle dorsiflexion strength, peak torque/body weight (%) | 0.000523 | 0.0000334, 0.00101 | 0.037* |
| AbH CSA (per 100 mm²) | −0.00132 | −0.0138, 0.0112 | 0.835 |
| FHB CSA (per 100 mm²) | −0.0147 | −0.0255, −0.00381 | 0.008* |
| FDB CSA (per 100 mm²) | 0.00174 | −0.0121, 0.0156 | 0.805 |
| Limb × (HVA+HIA) | −0.000489 | −0.00165, 0.000671 | 0.405 |

Values are fixed-effect estimates (β) with 95% confidence intervals from the sensitivity linear mixed-effects model restricted to athletes participating in sports involving frequent jumping, landing, cutting, pivoting, or rapid deceleration. The included sports were badminton, basketball, field hockey, gymnastics, handball, rugby, soft tennis, tennis, track and field, and wrestling. The model used the same fixed effects and repeated structure as the primary model. CSA coefficients are expressed per 100 mm² increase. P values are from Type III tests of fixed effects. *p < 0.05. GP, growth plate; BMI, body mass index; HVA, hallux valgus angle; HIA, hallux interphalangeal angle; AbH, abductor hallucis; CSA, cross-sectional area; FHB, flexor hallucis brevis; FDB, flexor digitorum brevis.
